# Artificial intelligence to meet the psychosocial needs of cancer patients: a systematic review of randomized controlled trials

**DOI:** 10.1590/1806-9282.20251636

**Published:** 2026-06-15

**Authors:** Nevra Didem Kılınç, Fahriye Oflaz

**Affiliations:** 1 Koç University, Institute of Health Sciences – Istanbul, Türkiye.; 2 Istanbul Arel University, Department of Nursing – Istanbul, Türkiye.

## INTRODUCTION

Cancer remains one of the leading causes of morbidity and mortality worldwide, and beyond its physical burden, the disease imposes considerable psychosocial challenges^1^. Patients frequently experience anxiety, depression, distress, and impaired quality of life throughout the diagnostic and treatment trajectory^2^. Psychosocial support is therefore recognized as an essential component of comprehensive cancer care^3^. However, access to such support is often limited due to shortages of mental health professionals, financial and institutional barriers, and the stigma still associated with seeking psychological help^4^.

In recent years, artificial intelligence (AI) has emerged as a promising tool to expand the reach of supportive care^5^. AI-powered interventions such as chatbots, mobile applications, and digital therapeutics can provide personalized, scalable, and cost-effective psychosocial support^6,7^. These tools represent a technological spectrum ranging from simple rule-based algorithms to advanced generative models^5^. Crucially, the literature frequently conflates basic automation with genuine machine learning (ML), creating conceptual heterogeneity that complicates interpretation.

Although individual trials suggest potential benefits, no systematic synthesis of randomized evidence has yet clarified the effectiveness. These gaps highlight the importance of systematically reviewing and evaluating AI-based interventions to determine their effectiveness in improving psychosocial well-being among cancer patients.

## METHODS

### Protocol and registration

This systematic review followed Preferred Reporting Items for Systematic Review and Meta-Analysis (PRISMA) guidelines and was registered in PROSPERO (CRD420251134621). Literature search, study selection, data extraction, and quality assessment were performed independently by two researchers (NDK and FO), with disagreements resolved through consensus meetings.

### Eligibility criteria

Studies were included if they met the PICOS framework: (P) patients diagnosed with cancer (any age, type, or stage); (I) AI-based interventions, defined operationally as tools utilizing ML (natural language processing, predictive modeling, and personalization based on behavioral data) or generative AI (large language models) to deliver psychosocial support; (C) usual care, standard psychosocial support, non-AI interventions; (O) psychosocial outcomes; (S) randomized controlled trials (RCTs) published in English. Exclusion criteria were unclear methodology, unavailable full text, observational designs, and simple rule-based algorithms or static digital health tools classified as non-AI digital interventions.

### Search strategy

A literature search was conducted in PubMed, Cochrane Library, Scopus, Web of Science, and CINAHL. Keywords included "cancer," "oncology," "artificial intelligence," "digital health," "mental health," "psychological intervention," "psychosocial intervention," "mental health support," "quality of life," "anxiety," and "depression."

### Data extraction

A standardized data extraction form was developed to collect key study characteristics. Extracted information included author details, publication year, country of study, study design, sample size, participant characteristics, intervention type and duration, comparison group, data collection period, and reported psychosocial outcomes. The data extraction process was conducted independently by two researchers (NDK and FO).

### Quality assessment

The methodological quality of the included RCTs was assessed independently by two researchers using the Cochrane Risk of Bias 2 (RoB 2)^8^ tool. Studies were evaluated across five domains: randomization process, deviations from intended interventions, missing outcome data, measurement of the outcome, and selection of the reported result, with overall judgments assigned accordingly. Disagreements between researchers were resolved by discussion.

## RESULTS

### Search results

**Figure 1:**
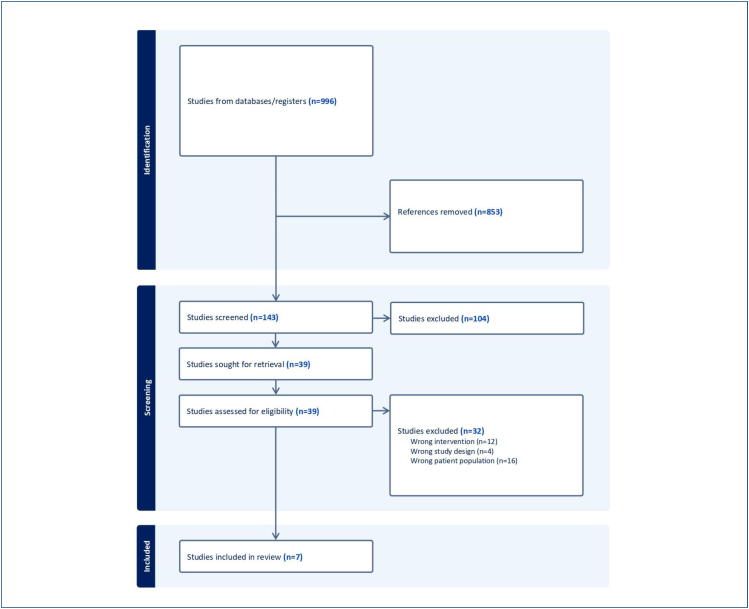
PRISMA flowchart.

A search of five databases revealed a total of 996 relevant articles. After removing duplicates, 143 studies were selected for full-text analysis, and only seven^9-15^ met the selection criteria for inclusion in this review (Table 1). The detailed PRISMA flowchart is shown in Figure 1.

**Table 1:** Characteristics of included studies.

Study, country	AI intervention type	Population intervention/control	Target outcome	Duration	Measurement tools
Akdogan et al.^9^ Türkiye	ChatGPT-based digital counseling	Chemotherapy-naïve cancer patients 75/75	Anxiety, depression	Duration not specified; 3 time points	HADS
Jiang et al.^10^, China	AI-driven mobile app (WeChat Mini Program)	Young breast cancer survivors 60/55	Psychological symptoms, self-efficacy, and social support	3-month intervention	MSAS-SF, CBI-B, SSRS, FACT-B
Kamdar et al.^11^, USA	ePAL AI pain app	Metastatic solid-organ cancer patients 56/56	Pain severity, attitudes	8-week intervention	BPI, BQ-II, GAD-7, FACT-G, PHQ-8
Lee et al.^12^, South Korea	Chatbot+video	Breast cancer patients 73/72	Anxiety	Duration not specified; 3 time points	APAIS, STAI, LASA
Schmitz et al.^13^, Germany	Nurse AMIE virtual assistant (Alexa/Echo Show)	Metastatic breast cancer 21/21	Distress, quality of life, fatigue	6-month intervention	SF-36 Quality of Life Survey, BPI, SF-36 Pain Subscale, Sleep Disturbance Scale, SF-36 Vitality Subscale, Distress Thermometer
Shaban et al.^14^, Egypt	AI-powered chatbot	Breast cancer patients 61/61	Knowledge, empowerment, attitude	Duration not specified; 2 time points	Knowledge Assessment, AI Support and Empowerment, Likert Scale for Attitude Towards AI
Springer et al.^15^, Germany	Mika app-based digital therapeutic	All tumor types 99/119	Distress	12-week intervention	NCCN, HADS, FACIT-F, CGI-I

APAIS: Amsterdam Pre-operative Anxiety and Information Scale; BPI: Brief Pain Inventory Short Form Composite Severity Score; BQ-II: Barriers Questionnaire-II; CBI-B: Cancer Behavior Inventory-Brief Version; CGI-I: Clinical Global Impression-Improvement Scale; FACIT-F: Functional Assessment of Chronic Illness Therapy-Fatigue; FACT-B: Functional Assessment of Cancer Therapy-Breast; FACT-G: Functional Assessment of Cancer Therapy; GAD-7: Generalized Anxiety Disorder; HADS: Hospital Anxiety and Depression Scale; LASA: Linear Analog Scale Assessment; MSAS-SF: Memorial Symptom Assessment Scale-Short Form; NCCN: National Comprehensive Cancer Network Distress Thermometer; PHQ-8: Patient Health Questionnaire-8; SSRS: Social Support Rating Scale; STAI: Korean Short Form of the State-Trait Anxiety Inventory.; SF-36: The 36-Item Short Form Health Survey.

### Quality assessment results

**Figure 2:**
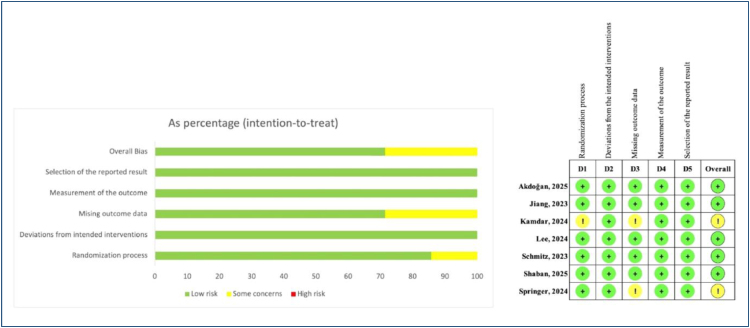
Risk of bias assessment (Risk of Bias 2).

The risk of bias assessment indicated that most included trials were judged to be at low risk across all domains (Figure 2). The majority were rated overall as low risk of bias, while two trials were assessed as having some concerns due to issues related to baseline imbalances, missing outcome data, or attrition linked to higher baseline distress.

### AI-based intervention results

AI-based methods in cancer care aim to enhance education, symptom management, and psychosocial support. Rule-based chatbots evaluated by Lee et al. and Shaban et al. provided information during radiotherapy or breast cancer management and reduced anxiety in some subgroups. A more advanced ChatGPT-4.0-based counseling intervention^9^ significantly reduced anxiety and depression before chemotherapy, emphasizing the need for expert validation. In a study by Jiang et al., it has been reported that "AI-TA" mobile app delivered personalized content, symptom tracking, and social support, improving psychological symptoms and self-efficacy. In an advanced cancer study by Kamdar et al., the ePAL app used AI to triage patient-reported outcomes, reducing pain scores and hospitalizations. Schmitz et al. tested the Nurse AMIE voice assistant, which showed high acceptability but no significant symptom effects. A study by Springer et al. reported that Mika app tailored support to cancer type, treatment stage, and user behavior, leading to reductions in distress, depression, anxiety, and fatigue. Only Jiang et al. explicitly applied a theoretical framework (person-centered care), while two studies incorporated therapeutic models like cognitive behavioral therapy and mindfulness-based stress reduction directly into the intervention content. Collectively, these approaches demonstrate AI's potential to deliver accessible, personalized psychosocial care, though expert oversight remains essential, especially for generative AI.

### Psychosocial outcomes

Lee et al. tested a chatbot and video education during radiotherapy in breast cancer patients; while no overall effect was observed, younger patients (age <50 years) showed reduced anxiety. Akdogan et al. demonstrated that a ChatGPT-based counseling tool significantly reduced pre-chemotherapy anxiety and depression. Jiang et al. reported that the AI-TA mobile app improved psychological symptoms, self-efficacy, and social support in young breast cancer survivors. Kamdar et al. found that the ePAL app lowered pain scores and reduced pain-related hospitalizations in advanced cancer. Schmitz et al. showed that the Nurse AMIE voice assistant achieved high acceptability and satisfaction, though without significant effects on distress, symptoms, or quality of life. Shaban et al. observed that an AI chatbot significantly increased knowledge, empowerment, and positive attitudes toward AI in breast cancer patients. Springer et al. demonstrated that the Mika therapeutic app reduced distress, depression, anxiety, and fatigue in cancer patients.

## DISCUSSION

This systematic review represents one of the first comprehensive syntheses of RCTs examining the effectiveness of AI-based interventions in addressing the psychosocial needs of cancer patients. The findings indicate that such digital tools show particularly promising effects in reducing anxiety and distress, while the evidence regarding depression and overall quality of life remains more limited and heterogeneous. A recent systematic review and meta-analysis demonstrated that digital psychosocial interventions (non-AI specific) significantly improved quality of life and produced moderate reductions in anxiety and depression among cancer patients, providing preliminary evidence for their integration into supportive care^16^. The trials also consistently reported high levels of acceptability, feasibility, and patient satisfaction, with interventions demonstrating the capacity to enhance knowledge, self-efficacy, and patient engagement.

International cancer guidelines, including the NCCN Distress Management Guidelines^17^, ESMO Supportive Care Recommendations^18^, and the ASCO Survivorship Guidelines^19^, emphasize psychosocial care as an integral component of comprehensive oncology. These guidelines advocate for routine distress screening and timely psychosocial interventions for all patients. However, the translation of these recommendations into routine clinical practice remains limited due to barriers such as shortages of mental health professionals, institutional and financial constraints, and the persistent stigma associated with seeking psychological support^20^. The findings of this review suggest that AI-based interventions offer a tangible mechanism to operationalize these recommendations. For instance, the automated triage algorithms evaluated in this review^11^ directly address the guideline requirement for "timely intervention" by identifying high-risk symptoms in real time, effectively bypassing delays associated with traditional referral pathways. Similarly, the digital therapeutic approaches offering on-demand coping strategies^15^ provide a scalable solution to the "routine support" advocated by ASCO, particularly for patients facing access barriers or stigma. Within this context, AI-based interventions, ranging from chatbots and mobile applications to digital therapeutics, offer accessible, scalable, and personalized solutions that could help bridge the gap between guideline recommendations and real-world care delivery^21^.

The findings suggest that AI interventions serve well as flexible adjuncts to conventional care. However, they introduce ethical risks including algorithmic bias^22^, generative AI "hallucinations"^9^, and data privacy concerns^23^. Furthermore, technological disparities may exclude older or lower-socioeconomic patients^24^. Consequently, large-scale, longitudinal studies are essential to establish the sustained effectiveness and safe integration of these tools into oncology practice.

## CONCLUSION

AI-based interventions show promise for alleviating anxiety and distress, though efficacy varies. Their scalability offers a compelling opportunity to bridge service gaps and empower patients. However, integration faces significant challenges, including algorithmic bias, data privacy concerns, and digital literacy barriers. Consequently, AI should be developed as a scalable adjunct to, rather than a replacement for, professional human support. Future large-scale, longitudinal research must prioritize evaluating safety and ethical frameworks alongside clinical outcomes to ensure AI becomes a safe and integral component of comprehensive oncology care.

### Limitations

Several limitations define this review. The small number of available RCTs, combined with significant heterogeneity in intervention types and outcome measures, restricted generalizability and precluded quantitative synthesis. Most interventions lacked a theoretical framework and assessed psychosocial outcomes only as secondary endpoints. Data on long-term sustainability remain scarce, with few studies providing extended follow-up. Finally, the restriction to English-language publications and major biomedical databases, excluding specialized technology repositories due to access constraints, may have introduced selection bias.

## Data Availability

The datasets generated and/or analyzed during the current study are available from the corresponding author upon reasonable request.
